# Unwanted pregnancy, mental health and abortion: untangling the evidence

**DOI:** 10.1186/1743-8462-5-2

**Published:** 2008-04-29

**Authors:** Judith M Dwyer, Terri Jackson

**Affiliations:** 1 Department of Health Management, Flinders University, Adelaide, Australia; 2 Australian Centre for Economic Research on Health, University of Queensland, Brisbane, Australia

## Abstract

Abortion policy is still contentious in many parts of the world, and periodically it emerges to dominate health policy debates. This paper examines one such debate in Australia centering on research findings by a New Zealand research group, Fergusson, Horwood & Ridder, published in early 2006. The debate highlighted the difficulty for researchers when their work is released in a heightened political context. We argue that the authors made a logical error in constructing their analysis and interpreting their data, and are therefore not justified in making policy claims for their work. The paper received significant public attention, and may have influenced the public policy position of a major professional body. Deeply held views on all sides of the abortion debate are unlikely to be reconciled, but if policy is to be informed by research, findings must be based on sound science.

## The policy questions

The deeply held beliefs on all sides of the debate about health services for women with unplanned pregnancies make it one of the most volatile and unpredictable of health policy issues. The uneasy political compromise that holds in Australia and New Zealand is one which allows women access to abortion on the basis of the common law or statute, but retains a criminal offense of abortion [1,2]. This arrangement is periodically challenged by those who believe that termination of pregnancy should never be allowed, or allowed only in the narrowest of circumstances.

**Figure 1:**
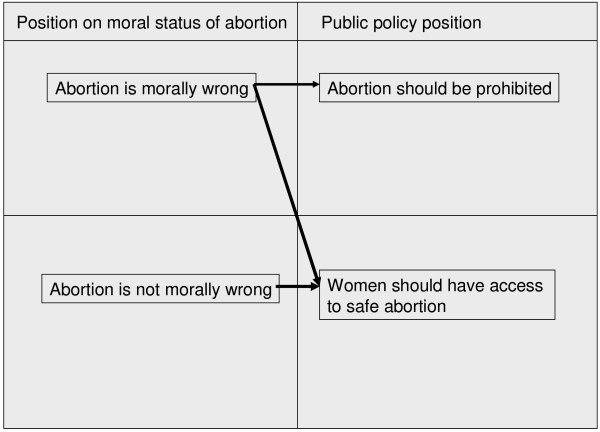
Views on moral status and public policy for abortion.

The policy logic of the prohibitionist view is that women and their health care providers should be prevented from having or providing abortions, by means of criminal sanctions, because abortion is morally repugnant. However, strong majorities in Australia [3] and New Zealand take the view that women should have access to safe abortion services. It is clear from the research on public opinion that people differentiate between the moral and public policy questions. The majority view on the public policy question (in support of safe service provision) is held by people who hold varying personal positions on the morality of abortion, as modelled (See Figure 1).

The logic of the majority view in support of provision of abortion services is based on two main foundations. The first is that women as persons have the moral capacity (and in democracies the right as citizens) to make decisions about their reproductive lives, including their pregnancies. The second is the 'harm minimisation' position that the health and equity impacts of prohibitionist policies are unacceptable.

In any case, the public policy debate is centred on the issue of access to services. While moral wrongness is the *basis *of the prohibitionist position, prohibition (or restriction) of access to services is the policy *outcome *that is sought. Similarly, the public policy goal of the 'pro-choice' position is access to safe affordable abortion services. This goal usually proceeds from the view that the moral decision is for the woman to make, and may or may not be based on a positive moral view of abortion. The long-standing prohibitionist position is that the distinction between the moral and public policy positions should not be made – that is, that establishing moral wrongness is enough to establish the case for criminal sanctions.

However, in recognition of the stability of the majority view, the prohibitionist arguments have shifted in recent years towards a position that abortion is bad for women's health, and women should therefore be protected from it by prohibition. This has resulted in the recent controversies over abortion and breast cancer (research evidence reviewed by Beral, Bull, Doll et al [4]) and the continuing debate about women's exposure to grief and regret as a result of terminating a pregnancy (reviewed by Bonevski and Adams[5]). The 'protection by prohibition' stance may also be aimed at influencing the views of those who support access to abortion while considering it to be morally wrong.

Most participants in the debate would agree that young pregnant women are 'vulnerable' to emotional and mental health problems. Differences arise, however, as to the greatest risk factors facing them. While prohibitionists emphasise the risks of negative outcomes of abortion, pro-choice groups emphasise the risks of unsafe abortions; of women being denied autonomy in making reproductive decisions (in particular being coerced by parents, partners, etc. to either continue or terminate a pregnancy); and the negative effects of exposure to judgmental behaviour or shaming by health care providers. All women, including those whose mental health is compromised for any reason – pre-existing mental illness, the stress of unwanted pregnancy, diagnosis of fetal problems in an otherwise wanted pregnancy, rape or other violence surrounding the conception – require appropriate care [6-8].

## The political context

The political context in Australia has been volatile in recent years, as the difficult processes involved in making non-surgical abortion methods available illustrate. The appointment of Tony Abbott as Australian Minister for Health in October 2003, and government control of the Senate after the October 2004 election, opened another period of political contestation about the availability of abortion services. Speaking in March 2004 the new Minister argued that the 'problem with the contemporary Australian practice of abortion is that an objectively grave matter has been reduced to a question of the mother's convenience...'[9]. Immediately following the 2004 election, a range of conservative politicians including Deputy Leader John Anderson and Senator-elect Barnaby Joyce advocated reopening the abortion debate [10].

Despite a Cabinet decision in early November 2004 that ruled out parliamentary consideration of the abortion issue [11], the retirement of Senator Brian Harradine meant that government deals previously struck with the conservative independent Senator to gain his support for legislation were no longer relevant. In particular, in 1996 Senator Harradine had negotiated special arrangements whereby the use of medical abortifacients including RU486 (also known as mifepristone) would be regulated directly by the Minister for Health rather than by the Therapeutic Goods Administration (TGA) [12].

Professor Caroline de Costa's call to regularise access to RU486 in the *Medical Journal of Australia *in October 2005 [13] reopened the debate, with early support from the president of the Australian Medical Association [14] and a number of female Coalition members of Parliament [15]. Early in 2006, debate on the drug RU486 was again taken up in the Australian Parliament on a private members' bill to repeal the special ministerial powers over regulation of the drug. In a 'conscience vote' allowed by all parties in the Senate and House of Representatives in February 2006, regulation of safety and efficacy was returned to the Therapeutic Goods Administration where all other pharmaceuticals are approved.

This example illustrates both the difficulty of applying normal regulation and standards to medical practice in termination of pregnancy and the volatile environment for research publication. Media attention to the Fergusson et al study [16], reporting an association between abortion and poor mental health status in young women, may have been amplified due to its timing (the quiet period of the southern hemisphere Christmas/summer shutdown). But, as the editor predicted in an accompanying editorial [17], it was in any case going to generate another outbreak of the abortion debate given the political context.

Researchers have very little control over the timing of publication of their results, and the coverage that was generated in Australia and New Zealand may not be what the researchers intended. However, the original paper explicitly claimed that its findings challenge the existing professional consensus that abortion is not the cause of significant harm to women's mental health. We argue below that the study does not support the authors' claims of policy relevance because of a fundamental design flaw.

## What the New Zealand study claims

The Fergusson et al paper [16] is one of many papers (including another on abortion and subsequent life outcomes [18]) emerging from a major longitudinal study of a cohort of 1265 children (630 girls) from birth to 25 years of age, the Christchurch Health and Development Study.

The longitudinal database used in the analysis incorporated measures recorded during the childhood of the young women surveyed, including family and socio-demographic background, childhood sexual or physical abuse, school achievement and behaviour, and measures of neuroticism. The analysis also made use of data collected when respondents were 15, the baseline for the pregnancy outcome study. These measures included self-esteem, onset of sexual activity, smoking, drinking and use of cannabis, and prior history of depression, anxiety disorders and/or suicidal thoughts.

Mental health outcome measures (major depression, anxiety disorder, suicidal thoughts, drug dependence and a count of mental health problems) were recorded for these women at ages 18, 21 and 25. For this study, the authors divided the sample into three groups: those who did not become pregnant during this period, those who had a baby, and those who had an abortion (74 young women in the 21–25 year age group). Univariate analysis of these variables demonstrated that 'troubles travel together'. Young women having abortions scored higher on most childhood and baseline mental health risk factors (Table 2, p20), particularly the subgroup of respondents who became pregnant and had an abortion between the age of 15 and 18 (Table 1, p19).

Risk ratios for poor mental health outcomes were adjusted for 'confounding pre-pregnancy factors, including social background, childhood and family history; mental health and personality factors.' (p 17). The omission of consideration of the wantedness of the pregnancy from this statement of research aims is significant.

The results show that young women who responded to pregnancy by having an abortion experienced higher rates of mental health problems both prior to (baseline measures at age 15) and following their pregnancies, and had higher incidence of various risk factors in their lives (such as history of sexual abuse) than young women who did not become pregnant. Young women who had babies fell somewhere in between these two groups on many of the measures. The authors claim that these results indicate 'a possible causal linkage between exposure to abortion and mental health problems' (p22).

The study has an exclusive focus on abortion, rather than on unwanted pregnancy, for reasons which are explained only in terms of the lack of available data (p22). It is clear that the authors did not design their data collection instruments to answer this research question. However, the discussion of the study's limitations acknowledges the question of wantedness as one of several 'other factors associated with the process of seeking and obtaining an abortion' (p22); and goes on to acknowledge that 'It is possible, therefore, that the apparent associations between abortion and mental health found in this study may not reflect *the traumatic effects of abortion per se *but rather other factors which are associated with the process of seeking and obtaining an abortion. For example, it could be proposed that our results reflect the effects of unwanted pregnancy on mental health rather than the effects of abortion per se on mental health.' (p22) (italics added). Despite this cautious wording, they go on to claim in both the conclusion and in the study's abstract that the current consensus among relevant professional bodies that the evidence does not indicate a causal relationship between abortion and mental illness is now in question.

The accompanying editorial [17] also acknowledges the importance of the question of wantedness: 'The key comparison is between women who carried unwanted pregnancies to term and those who had an abortion.' The editor goes on to assert that 'Fergusson and colleagues will be able to find out the answer to this question when next they interview their study participants', without acknowledging the known unreliability of post-hoc recollections of psychological states [19,20].

## Evidence-based policy or agenda-setting?

Policy discussion on health matters in recent times has applied the paradigm of 'evidence-based medicine' to a new construct: 'evidence-based policy' [21]. Its champions see the insertion of 'evidence' into political decision-making as a means toward more 'rational' outcomes. In the medical arena this is characterized by Rodwin as '...the hope that medical information will lead, without controversy or politics, to better medical care, and better health policy' [22]. To the extent that consensus can be achieved around a set of empirical facts, evidence-based policy may shift entrenched interests.

A longer-standing approach to policy development is broadly termed 'agenda setting.' As Stone [23] observes, questions for policy analysis are framed in political struggle, and political reasoning is always conducted as part of a struggle to control which images of the world govern policy. This has been highlighted in observations of the way in which communication media focus the attention of voters and decision-makers on particular issues and not others [24].

Both 'evidence-based policy' and 'agenda setting' are plausible ways of understanding the publication and response to the Fergusson paper. The Fergusson paper seeks to contribute evidence for policy, but we argue below that a problem in the study's research design means that it fails to do so, and has instead contributed only to agenda-setting.

## Evidence-based policy: 'People who take hangover remedies get headaches'...

The authors of this study are experts in the use of data to understand relationships between observed characteristics of a sample and a dependent variable of interest. Such multivariate models, however, are vulnerable to error when misspecified, particularly by omission of key covariates. For example, in investigating a potential relationship between gender and road accidents, it is necessary to control for the effects of age and kilometres driven. To make a case for causality, such studies must address several criteria [25], including the strength of the association, its consistency, specificity, temporality, biological gradient, plausibility and coherence.

For example, it would probably be possible to demonstrate that people who take hangover remedies are more likely to experience headaches than people who don't. It may also be the case that there are somewhat more headaches among those who drink (but don't take hangover medicines) than those who don't drink. However, this would not lead us to conclude that taking hangover medicines gives you headaches, and more so if you drink. This is because we know that drinking enough will give you a hangover, of which headache is a major symptom; and taking hangover treatments is a response. While the variables might both be correlated with headaches, the relationship is not causal.

On this analogy, the Fergusson et al study could be criticised because it takes the 'symptom' (abortion) to be the 'cause' of poorer mental health outcomes. If, however, we take into account whether the pregnancy is a wanted one or not, causation becomes more problematic. Abortion and unwanted pregnancy are highly correlated; and 'unwantedness' is the reason for most abortions (the important exception being those pregnancies aborted following a diagnosis of fetal abnormality or serious maternal illness or injury).

If it is the case that most of those who continued their pregnancies did so because the pregnancy was initially, or became, a wanted one, then the comparison in this study between those who continued their pregnancies and those who terminated them may in fact be a comparison of mental health outcomes for women who experienced a *wanted *pregnancy with those who experienced an *unwanted *pregnancy. The comparison between those who didn't get pregnant and those who aborted a pregnancy may tell us something about the stress of unwanted pregnancy, or the factors that make one more vulnerable to experiencing an unwanted pregnancy. The difference in mental health status could also be telling us something about the mental health outcomes of abortion, but as in the example of hangover remedies, by attributing causation to a response we may be missing the underlying causal relationships.

Unplanned or unwanted pregnancy can be a major crisis for women of any age, and there are several ways in which that crisis can be resolved, of which abortion is a major method. But the important point here is that many of the factors that make a pregnancy unwanted are in themselves stressful, and could perhaps be expected to be associated with lower scores on tests of mental health. The experience of sexual violence or coercion, lack of a supportive partner, poverty, and the factors that lead to lack of confidence in parenting ability are all challenging, but they are not controlled for in the current study.

There is evidence that poorer mental health status is associated with pregnancy and also with parenting in the teenage years. Kovacs, Krol and Voti [26] studied girls who were referred for depressive or conduct disorders and found that childhood or adolescent conduct disorders, but not depressive disorders, were associated with teenage pregnancy. In a large cohort study, Kessler et al [27] found that adolescent parenthood was associated with higher incidence of specific psychiatric disorders (prior diagnosis of anxiety, affective, addictive and conduct disorders). These studies and others indicate correlations between poorer prior mental health and both pregnancy and parenthood in the teenage years. The younger age at which the abortion group experienced unwanted pregnancy (Table 1, p19) may thus explain some of the differences in mental health outcomes.

The comparison with women who continued their pregnancies, in the absence of information about the circumstances and 'wantedness' of the pregnancy, sheds no light at all on the health effects of abortion. The only comparison that would do so is between women with unwanted pregnancies who aborted, and women with unwanted pregnancies who didn't abort (see, for example, the RCOG guidelines [6] and the study by Gilchrist et al. [28] which conclude that continuation of unwanted pregnancies is associated with poorer mental health outcomes). Taking account of Bradford Hill's 'consistency' criterion, much of the available scientific literature in fact rejects the 'abortion causes poor mental health' proposition; while it gives support for the proposition that unwanted pregnancy is a stressful experience [29].

## Agenda setting: Therefore hangover remedies are dangerous and should be withdrawn from sale..

The authors seem well aware of the weakness of their evidence, stating throughout the paper that they are investigating a 'linkage' between abortion and mental health outcomes, rather than causation. They acknowledge their study's failure to control for 'wantedness', but see it as a limitation rather than a fundamental design problem. Their discussion carefully acknowledges the omitted variables problem, but only after making the claim that the study results suggest 'a possible *causal linkage *between exposure to abortion and mental health problems' (p22; our emphasis).

They use this conclusion to argue that the current consensus on the psychological effects of abortion (that it is not, *per se*, a cause of mental illness) is challenged by their findings, and that the position of the American Psychological Association (which is consistent with the position of the Royal Australian and New Zealand College of Psychiatry [7]) should be reviewed. It is not known whether this argument was influential, but the APA statement has been withdrawn, pending updating [30].

If it were accepted, the authors' argument has potential impacts on the availability of abortion in Australia. The current 'legality' of abortion in many states rests on case law about medical necessity. That is, doctors must weigh up in each patient's case the potential harms and benefits of abortion compared with the harms and benefits of continuing the unwanted pregnancy. Credible evidence that abortion causes harms to women's mental health could well raise the implicit risk/benefit threshold in doctors' minds, particularly if tested in the courts. If credence were given to the results of this study in a case against a doctor charged with performing an 'illegal' termination of pregnancy, the political goal of reducing women's access to these services could be accomplished in the common-law jurisdictions through the courts, with minimal political pain.

## Conclusion

In entering a contested area of policy, Fergusson and colleagues may have understood their role as aiding the formulation of 'evidence-based' policy by adding empirical findings around which a new consensus about abortion policy could be built. They may not have understood how seriously the use of abortion as a convenient surrogate for 'unwantedness' compromises their conclusions about causation of mental health problems in young women. Unfortunately, the poor design of the study undermines the intent to inform policy with evidence.

It is inevitable that the reproductive health of women, and their capacity to make responsible decisions, will continue to be challenged and contested in an arid debate about 'rights' which is unhelpful and will probably never be resolved. At the same time, good quality research about reproductive health (especially the prevention of unwanted pregnancy), and about good practice in reproductive health care, is needed, as is resolution of the policy and legal context in which that care is provided.

## Authors' contributions

JD and TJ collaborated in the policy analysis and research for this study, and each participated in drafting. All authors read and approved the final manuscript.
